# Zinc Oxide Nanorod-Based Sensor for Precision Detection and Estimation of Residual Pesticides on Tea Leaves

**DOI:** 10.3390/mi16050569

**Published:** 2025-05-10

**Authors:** Baharul Islam, Rakesh A. Afre, Sunandan Baruah, Diego Pugliese

**Affiliations:** 1 Faculty of Engineering, Assam down town University, Sankar Madhab Path, Gandhinagar, Panikhaiti, Guwahati 781026, Assam, India; baharul.islam@adtu.in; 2 Department of Engineering Sciences, Zeal College of Engineering and Research, Survey No. 39, Narhe-Dhayari Road, Narhe, Pune 411041, Maharashtra, India; rakesh.afre@zealeducation.com; 3 Centre of Excellence in Nanotechnology, Assam down town University, Sankar Madhab Path, Gandhinagar, Panikhaiti, Guwahati 781026, Assam, India; 4 National Institute of Metrological Research (INRiM), Strada delle Cacce 91, 10135 Torino, Italy

**Keywords:** nanomaterial-based sensor, ZnO nanorod, pesticide detection, quinalphos, thiamethoxam, tea leaf safety

## Abstract

This study presents the development of a zinc oxide (ZnO) nanorod-based sensor for the detection and quantification of residual pesticides commonly found in tea plantations, with a focus on quinalphos and thiamethoxam. Exploiting the unique electrical characteristics of ZnO nanorods, the sensor exhibits high sensitivity and selectivity in monitoring trace levels of pesticide residues. The detection mechanism relies on measurable changes in electrical resistance when the ZnO nanorod-coated electrodes interact with varying concentrations of the target pesticides. A performance evaluation was carried out using water samples spiked with different pesticide concentrations. The sensor displayed distinct response profiles for each compound: a linear resistance–concentration relationship for quinalphos and a non-linear, saturating trend for thiamethoxam, reflecting their differential interactions with the ZnO surface. Statistical analysis confirmed the sensor’s reliability, reproducibility, and consistency across repeated measurements. The rapid response time and ease of fabrication underscore its potential for real-time, on-site pesticide monitoring. This method offers a promising alternative to traditional analytical techniques, enhancing food safety assurance and supporting sustainable agricultural practices through effective environmental surveillance.

## 1. Introduction

Nanomaterials, characterized by their distinctive physicochemical properties at the nanoscale, have significantly advanced sensor technology through their high surface-to-volume ratio, tunable electronic structures, and enhanced chemical reactivity. Among these, zinc oxide (ZnO) has emerged as a particularly promising semiconductor due to its wide band gap (3.37 eV), high exciton binding energy, and exceptional chemical and thermal stability—attributes that make it highly suitable for sensing applications [1]. A variety of synthesis techniques, including hydrothermal, sol–gel, chemical vapor deposition, and microfluidic-assisted methods [2], allow precise control over ZnO nanostructure morphology, which critically affects sensor’s performance. ZnO nanorods, in particular, offer a high density of active sites for analyte interaction and facilitate efficient charge transport, resulting in sensors with excellent sensitivity and selectivity. These properties have enabled the development of ZnO-based nanosensors for detecting a broad range of targets, including gases, biomolecules, and environmental contaminants such as pesticides [3]. Recent progress highlights the potential of ZnO nanorod sensors for rapid, real-time, and cost-effective pesticide residue detection, addressing pressing concerns in food safety and environmental monitoring [4].

Tea is one of the most widely consumed beverages worldwide, boasting a rich cultural heritage and significant economic value [5]. However, the exponential growth of the tea industry in recent decades has introduced new challenges in ensuring product quality and safety. In particular, the use of pesticides to safeguard tea crops from pests and pathogens has raised substantial concerns. While pesticides are indispensable for crop protection, their residual presence in the final tea products can lead to health hazards if they exceed established food safety limits [6,7]. Consequently, there is an urgent need for reliable, sensitive, and rapid methods to detect and quantify pesticide residues in tea, ensuring both consumer safety and compliance with food quality standards [8,9].

Traditionally, the detection and quantification of pesticide residues have relied on sophisticated analytical techniques such as gas chromatography (GC), gas chromatography–mass spectrometry (GC–MS), high-performance liquid chromatography (HPLC), and liquid chromatography–mass spectrometry (LC–MS) [10,11,12,13,14,15,16]. These methods have been widely adopted due to their excellent sensitivity, specificity, and ability to handle complex sample matrices. Despite these advantages, conventional approaches are often associated with high operational costs, lengthy sample preparation procedures, and a requirement for highly trained personnel. Moreover, their inherent limitations in terms of portability and real-time analysis restrict their widespread application in field-based or on-site monitoring.

To address these limitations, researchers have increasingly turned to nanotechnology-enabled sensors. ZnO nanorods, in particular, have drawn significant attention due to their exceptional electrical, optical, and surface properties. Their pronounced responsiveness to environmental stimuli renders them ideal for detecting trace contaminants such as pesticides. As a result, ZnO nanorod-based sensors offer a compelling alternative to traditional methods, combining sensitivity, speed, and operational simplicity to meet the growing demands of food safety and sustainable agriculture.

The sensor operates on the principle of monitoring the variation in electrical resistance induced by the adsorption of pesticide molecules on the surfaces of ZnO nanorods. When pesticide molecules interact with the ZnO surface, they induce charge transfer processes that modulate the carrier concentration within the nanorods. For instance, the adsorption of electron-donating species leads to an increase in the electron density within the conduction band (CB), thereby reducing the sensor’s resistance [17]. This change in resistance is directly correlated with the concentration of the pesticide, enabling quantitative analysis. A major advantage of the ZnO nanorod-based sensor is its ability to deliver high sensitivity and selectivity across a broad concentration range. In our study, the sensor was evaluated using water samples spiked with pesticide concentrations at 0.1, 0.25, 0.5, 0.75, and 1% (*v*/*v*) for both quinalphos and thiamethoxam. The sensor’s response to quinalphos was observed to be linear over the tested concentration range, with a clear proportional relationship between the concentration and the decrease in the sensor’s resistance. In contrast, the response to thiamethoxam exhibited a non-linear behavior, with the resistance decreasing progressively until reaching a saturation level at higher concentrations. This distinct difference in response not only underscores the sensor’s sensitivity but also its selectivity; the sensor is capable of distinguishing between different pesticide types based on their interaction mechanisms with the ZnO surface.

One of the critical challenges in sensor development is ensuring consistent performance, particularly in terms of repeatability and reproducibility. To address this, a series of controlled experiments using spiked samples and repeated measurements were conducted. Statistical analysis of the sensor’s performance across multiple trials confirmed that the ZnO nanorod-based sensor exhibits excellent repeatability, with minimal variation in its response to identical pesticide concentrations. This reliable performance is crucial for practical applications, where repeated on-site analyses are frequently required. The integration of ZnO nanorod-based sensors into portable and cost-effective monitoring systems presents a promising alternative to conventional analytical methods [15,17]. With rapid response times—in our experiments, the sensor’s response approached saturation within approximately 30 s after exposure to pesticides—the ZnO nanorod sensor offers the potential for real-time monitoring, facilitating immediate decision-making in agricultural and environmental safety contexts. Additionally, the low-cost and scalable nature of the sensor’s fabrication process further enhances its appeal for widespread deployment, particularly in resource-limited settings where expensive laboratory infrastructure may not be available [18,19].

Several key aspects underscore the novelty of the present study. First, a ZnO nanorod-based chemiresistive sensor was developed for the selective detection of quinalphos and thiamethoxam pesticides in tea cultivation environments—an application of particular importance due to the global consumption of tea and associated health concerns arising from pesticide residues. Second, the sensor exhibits distinct response mechanisms for different pesticide classes: a linear decrease in resistance for quinalphos, attributed to electron density modulation in the ZnO CB, and a non-linear, saturation-type response for thiamethoxam, resulting from the limited availability of reactive adsorption sites. This differentiated behavior enables both detection and discrimination between pesticide types. Third, the sensor demonstrates rapid response times (approximately 30 s), along with high repeatability and reliability, rendering it suitable for real-time monitoring applications. Finally, the proposed approach integrates the advantages of nanomaterial-based sensing—such as high surface area and enhanced sensitivity—with cost-effective fabrication techniques, thereby addressing the demand for accessible and efficient monitoring tools in agricultural settings where advanced analytical infrastructure may be lacking.

## 2. Materials and Methods

### 2.1. Chemicals

All chemicals used in this work were of analytical grade and employed without further purification. Zinc acetate dihydrate (Zn(CH_3_COO)_2_·2H_2_O), zinc nitrate hexahydrate Zn(NO_3_)_2_·6H_2_O with 99% purity, and ethanol (C_2_H_5_OH) with 99.5% purity were purchased from Merck (Guwahati, Assam, India). Sodium hydroxide (NaOH) with ≥98% purity was acquired from Sigma-Aldrich (Guwahati, Assam, India). Hexamethylenetetramine (C_6_H_12_N_4_) with 99.5% purity was purchased from Carlo Erba (Guwahati, Assam, India). The quinalphos (C_12_H_15_N_2_O_3_PS) and thiamethoxam (C_8_H_10_ClN_5_O_3_S) pesticides were obtained from Jallan Golaghat Tea Co. (Golaghat, Assam, India), and used as received. Pesticide solutions were prepared by diluting the stock solutions into various concentrations using deionized (DI) water.

### 2.2. Fabrication of the Sensing Electrode

DipTrace^®^ software (version 4.3.0.5) was used to design the electrode schematic, which was then imported into a printed circuit board (PCB) layout editor. To prepare the PCB for the etching process, masking tape was applied to shield designated areas. During sensor fabrication, the electrode pattern was defined on a copper substrate using a masking technique, followed by the deposition of the sensing material. The final sensor measured 15.3 × 6.8 mm^2^, with an inter-electrode gap of 0.2 mm and electrode widths of 0.4 mm.

### 2.3. Synthesis of Seed ZnO Nanoparticles

A solution of 4 mM zinc acetate dihydrate was prepared in 20 mL of ethanol under vigorous stirring at 50 °C. Subsequently, the solution was diluted with an additional 20 mL of fresh ethanol and allowed to cool to ambient temperature. Following this, 20 mL of 2 mM sodium hydroxide in ethanol was added dropwise under continuous stirring. The resulting mixture was maintained in a temperature-controlled water bath at 60 °C for 2 h, after which it was cooled to room temperature [20]. Finally, a dip-and-dry process was employed to deposit the ZnO nanoparticles onto pre-designed and fabricated electrodes. After deposition, the electrodes were heat-treated in a hot air oven at 120 °C for 5 h to ensure proper fixation of the nanoparticles on the electrode surfaces and to remove any residual organic materials [20].

### 2.4. Growth of ZnO Nanorods on Seeded Substrate

**Figure 1:**
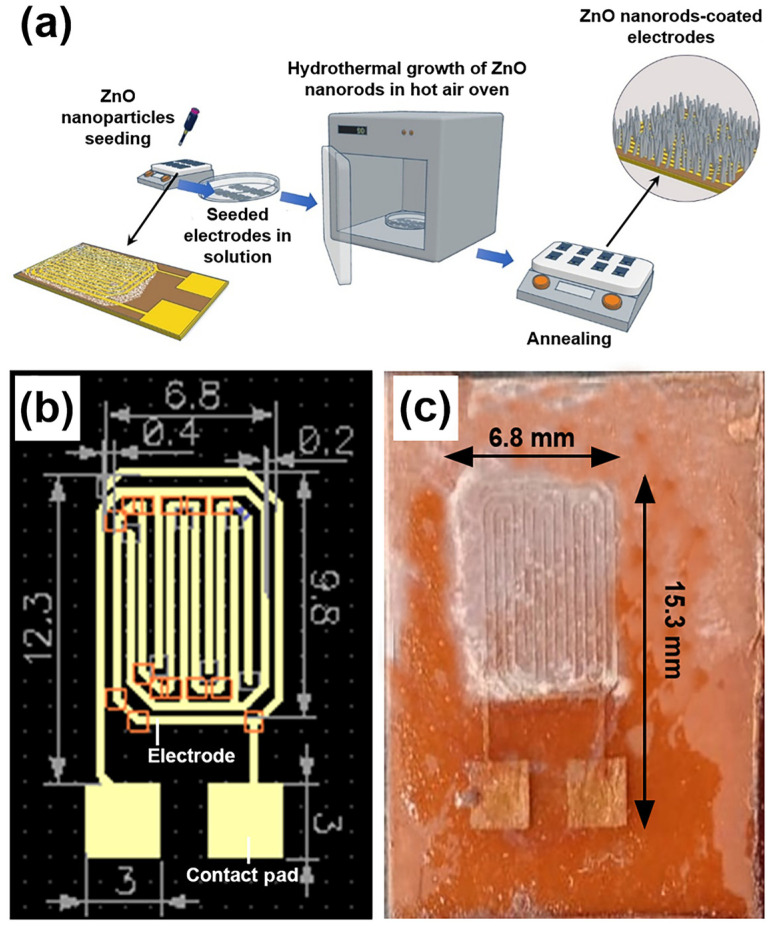
(**a**) Schematic illustration of the ZnO nanorod sensor fabrication process; (**b**) layout of the designed sensing electrode using DipTrace^®^ software; and (**c**) photograph of the fabricated ZnO nanorod-based sensor.

An equimolar solution of zinc nitrate hexahydrate and hexamethylenetetramine was prepared at a concentration of 15 mM for the growth of ZnO nanorods on the seeded electrode. The growth of ZnO nanorods on the seeded copper substrate was carried out for 30 h. During this process, the substrate was immersed in the equimolar solution, which was replaced every 5 h. The growth was performed in a hot air oven maintained at 90 °C. After 30 h, the samples were rinsed with DI water and dried at 120 °C for 1 h [21,22,23]. The fabrication process of the ZnO nanorod-based sensor is schematically illustrated in Figure 1a, while Figure 1b and Figure 1c show the layout of the designed sensing electrode and a photograph of the final fabricated sensor, respectively.

### 2.5. Sample Preparation

Samples for testing were prepared using DI water containing quinalphos at concentrations of 0.1, 0.25, 0.5, 0.75, and 1% (*v*/*v*). Similarly, thiamethoxam samples were prepared with concentrations ranging from 0.1 to 1% (*v*/*v*) using DI water. To evaluate the efficacy of the sensor, the pesticide solutions were sufficiently diluted in DI water.

### 2.6. Characterization

Transmission electron microscopy (TEM) was performed in a TECNAI G2 20 S-TWIN (FEI, Hillsboro, OR, USA) at 200 kV to determine the size of the ZnO nanoparticles and to confirm their crystalline nature. Scanning electron microscopy (SEM) images of the ZnO nanoparticles and nanorods were obtained using a JSM 6390LV (JEOL Ltd., Tokyo, Japan) operated at 5 kV.

X-ray diffraction (XRD) analysis was carried out using a Bruker D8 Discover diffractometer (Bruker, Billerica, MA, USA) equipped with a CuKα radiation source (*λ* = 1.54056 Å). The measurements were performed over a 2*θ* range of 5 to 70°, at a scanning rate of 0.02°/s, with an excitation voltage of 25 kV, and a current of 40 mA.

The absorption spectra of both the ZnO nanostructures were acquired in the wavelength range of 200–1000 nm using the Ultraviolet–Visible (UV–Vis) spectrophotometer FLAME-S-XR1-ES (Ocean Insight, Orlando, FL, USA).

A digital multimeter (HTC DM-97, HTC Instruments, Mumbai, India) was used to record the current and voltage measurements, enabling the evaluation of the sensor’s current–voltage (*I*–*V*) characteristics. A direct current (DC) voltmeter (Falcon PS-303S, Falcon Electro-Tek Pvt Ltd., New Delhi, India) was employed to supply the voltage to the sensor. During sensor operation, a visible light source was utilized to simulate ambient illumination conditions. Specifically, a 20 W light-emitting diode (LED) tube light manufactured by Havells India Ltd. (Noida, Uttar Pradesh, India) served as the illumination source. The light intensity at the sensor surface was measured to be approximately 1.403 kilolux using a digital lux meter (model LX-1010B, Mextech Technologies India Pvt. Ltd., Mumbai, Maharashtra, India). The LED emits broadband visible light covering the 400–700 nm range, consistent with the requirements for photoactivation of ZnO-based sensors.

## 3. Results

### 3.1. TEM, SEM, and XRD Characterization of ZnO Nanoparticles and Nanorods

**Figure 2:**
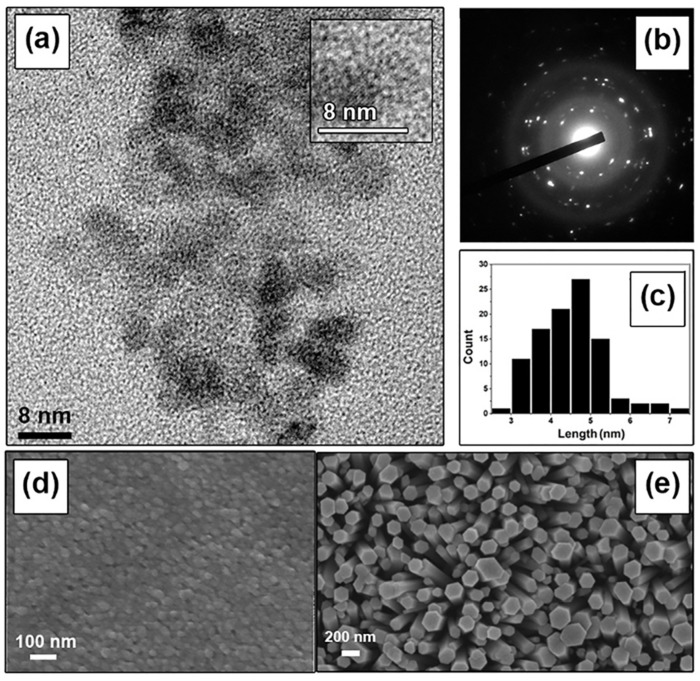
(**a**) Low-resolution TEM micrograph of ZnO nanoparticles; (**b**) electron diffraction pattern of ZnO nanoparticles; (**c**) size distribution histogram (a total of 100 particles were analyzed for size distribution using ImageJ^®^ software (version 1.51k)); (**d**) SEM images of seed ZnO nanoparticles; (**e**) top-view SEM images of ZnO nanorods.

Figure 2a,b present TEM images of the synthesized ZnO nanoparticles. The low-resolution TEM image in Figure 2a reveals that the nanoparticles are predominantly spherical with diameters ranging from 4 to 5 nm, while the electron diffraction pattern in Figure 2b confirms their crystallinity. Analysis of the TEM images indicates that the particle size distribution is largely within the 4–5 nm range (Figure 2c). Figure 2d,e display SEM micrographs of the synthesized ZnO nanoparticles and ZnO nanorods grown on the Cu interdigitated electrodes, respectively. During the seeding procedure, the ZnO nanoparticles agglomerated to form larger clusters with sizes of approximately 30–40 nm. This agglomeration is attributed to the surface tension of the ethanol solvent, which draws the particles together during the drying process [24]. The average size of these agglomerates is measured to be approximately 40 nm. Furthermore, the vertically oriented nanorods exhibit an average width of 225 nm and a length of 4 µm, as shown in Figure 2e.

**Figure 3:**
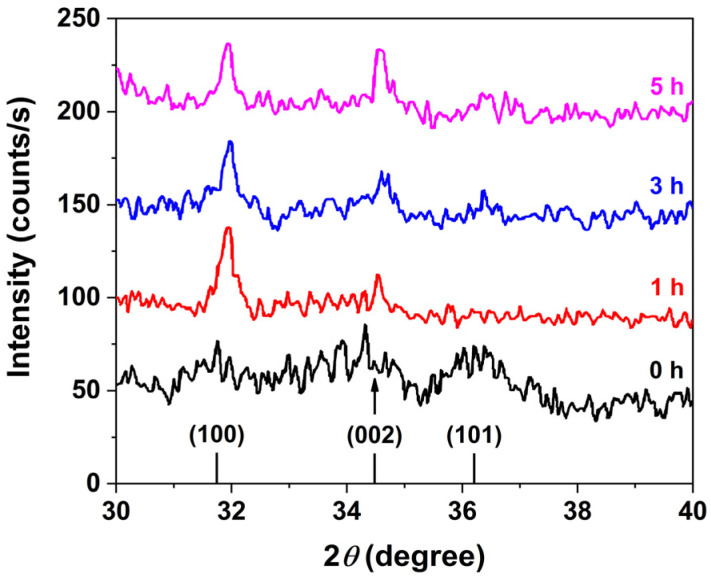
XRD patterns of ZnO nanorods synthesized at different growth times (0, 1, 3, and 5 h), showing preferential orientation along the [0001] direction.

Powder XRD analysis confirmed that the ZnO nanorods crystallize in the wurtzite phase, in agreement with the standard Joint Committee on Powder Diffraction Standards (JCPDS) pattern for ZnO (file no: 043-0002), as shown in Figure 3. A comparison of XRD patterns corresponding to different growth durations reveals a progressive enhancement in the (002) peak intensity, indicative of preferential anisotropic growth along the [0001] crystallographic direction. This behavior is attributed to the higher mobility of Zn^2+^ ions compared to the bulkier hexamine molecules. Initially, the more agile Zn^2+^ ions promote rapid, isotropic nucleation and growth of ZnO. As the reaction proceeds, long-chain hexamine molecules selectively adsorb onto the nonpolar facets of the wurtzite ZnO crystals, effectively blocking further access of Zn^2+^ ions to these surfaces. This facet-selective passivation restricts further growth to the polar c-axis, resulting in the elongation of ZnO nanorods along the [0001] direction.

### 3.2. UV–Visible Spectroscopy of ZnO Nanoparticles and Nanorods

**Figure 4:**
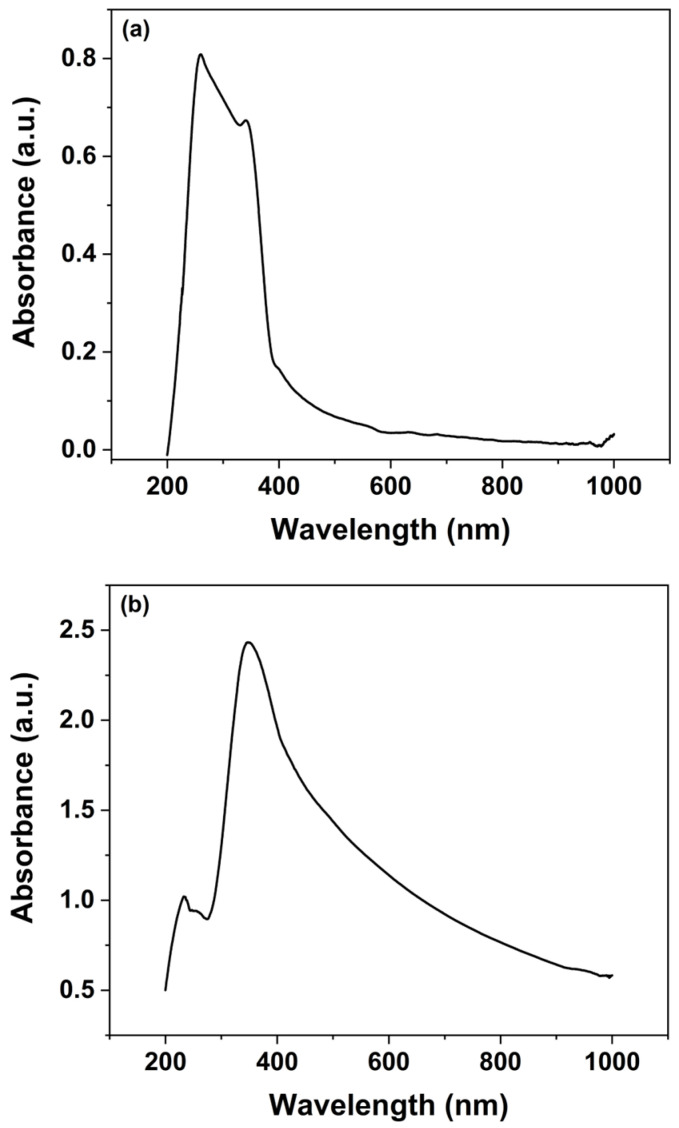
Absorbance spectra of the ZnO: (**a**) nanoparticles and (**b**) nanorods.

UV–Vis spectroscopy was employed to investigate the optical characteristics of both the synthesized ZnO nanoparticles and the ZnO nanorods grown over 30 h, as illustrated in Figure 4. The data obtained from these measurements provide valuable insights into the electronic transitions and band gap properties of the ZnO nanomaterials. The analysis of absorbance was conducted using the Beer–Lambert law, which is represented by the following equation:

(1) $$Absorbance=\log\left(\frac{I_{0}}{I}\right)=\varepsilon cL,$$

where ε represents the molar absorptivity, c denotes the concentration of the absorbing species, and L stands for the path length of light through the medium. In this formula, $I_{0}$ and I represent the intensities of the incident and transmitted light, respectively, allowing absorbance to be calculated from their ratio.

For the UV–Vis analysis, the spectrophotometer operated over a wavelength range of 200 to 1000 nm, providing a broad spectral response. Analysis of the spectra indicates that the ZnO nanoparticles exhibit maximum UV absorbance in the range of 230–380 nm, which is characteristic of ZnO’s intrinsic band gap absorption (Figure 4a). In contrast, the ZnO nanorods show a pronounced absorbance peak near 400 nm (Figure 4b). The observation of UV absorbance confirms the presence of ZnO in the nanorods, while the additional absorption in the visible range reveals enhanced light-interaction properties that are critical for sensor’s functionality. The presence of visible spectrum absorption not only underscores the optical quality of the ZnO nanorods but also suggests that these materials can be effectively employed in sensors operating under visible light conditions. This is particularly advantageous for the detection of pesticides, many of which exhibit absorption bands in the visible region.

The dual optical response of the ZnO nanomaterials enhances the sensor’s capabilities by enabling the detection of pesticides through changes in the visible light spectrum, thereby broadening its potential application in ambient conditions and real-time monitoring. Overall, the UV–Vis spectroscopy analysis corroborates the structural and optical integrity of the synthesized ZnO nanomaterials, affirming their suitability for integration into sensor platforms aimed at environmental and food safety applications.

**Figure 5:**
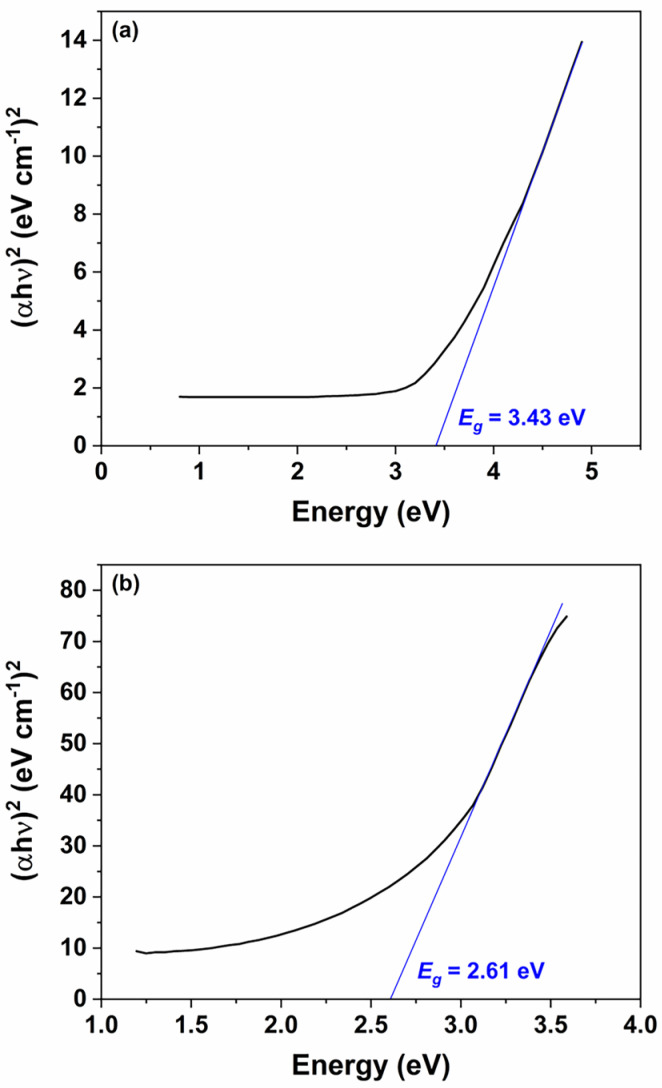
Tauc plots of the ZnO: (**a**) nanoparticles and (**b**) nanorods.

Figure 5a,b present the Tauc plots for the ZnO nanoparticles and nanorods, respectively. The estimated optical band gaps are 3.43 eV for the nanoparticles and 2.61 eV for the nanorods. The observed reduction in the band gap for the nanorods can be attributed to the increased presence of intrinsic defects, such as oxygen vacancies and zinc interstitials, that arise during the transition from spherical seed particles to the anisotropic nanorod morphology. This narrowing of the band gap enables effective utilization of visible light, as 2.61 eV corresponds to a wavelength of approximately 475 nm, which lies within the visible region of the electromagnetic spectrum.

### 3.3. Hydrolysis and Reaction Mechanism

**Figure 6:**
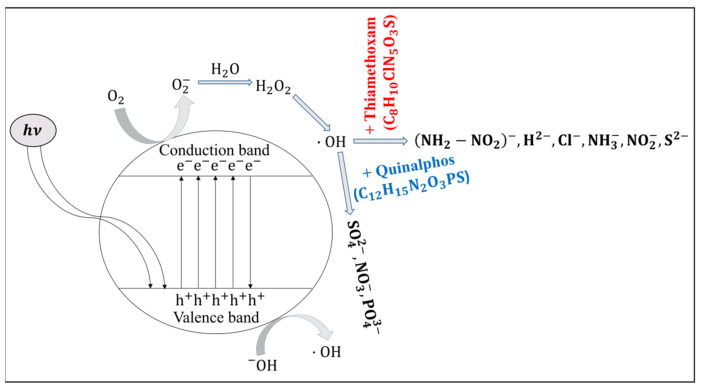
Proposed degradation pathway of quinalphos and thiamethoxam pesticides.

When light interacts with ZnO, photons with energy equal to or greater than the band gap excite electrons from the valence band (VB) to the CB, leaving behind positively charged holes. This photoexcitation process generates electron–hole pairs (Equation (2)), which contribute to the material’s photoconductivity—a key factor in the sensor’s performance. Figure 6 schematically illustrates this mechanism, showing the movement of electrons into the CB and the simultaneous formation of electron vacancies (holes) in the VB [25]. These charge carriers enhance the electrical response of ZnO under illumination, thereby improving the sensitivity of devices that rely on these optical and electrical transitions.

(2) $$ZnO+h\nu \to e_{CB}^{-}+h_{VB}^{+},$$

where h is the Planck’s constant (h = 6.626 × 10^–34^ J·s), ν is the frequency of the radiation, $e_{CB}^{-}$ are the CB electrons, and $h_{VB}^{+}$ are the VB holes.

When ZnO nanorods are irradiated with light whose energy exceeds the material’s band gap (3.37 eV), electrons are excited from the VB to the CB, generating free carriers. These photoexcited electrons can interact with molecular oxygen ($O_{2}$) adsorbed on the ZnO surface, reducing oxygen to form superoxide radical anions ($O_{2}^{-}$ or ˙$O_{2}^{-}$) (Equation (3)). The formation of these reactive oxygen species is a critical step in the sensor’s operational mechanism, as the superoxide radicals tend to trap free electrons, thereby modulating the electrical conductivity of the nanorods. This change in conductivity due to surface reaction kinetics not only enhances the photoresponse of the ZnO sensor but also facilitates subsequent interactions with target analytes, such as pesticides. Additionally, the generation of ˙$O_{2}^{-}$ contributes to a series of redox reactions at the sensor’s interface, further influencing the sensor’s sensitivity and selectivity under illumination.

(3) $$O_{2}+e_{CB}^{-}\to O_{2}^{-}$$

This $O_{2}^{-}$ radical, when hydrolyzed, it generates $H_{2}O_{2}$ and $O_{2}$:

(4) $$O_{2}^{-}+H_{2}O\to H_{2}O_{2}+O_{2}$$

As this reaction occurs in the presence of light, $H_{2}O_{2}$ can absorb the energy from light and undergo photochemical decomposition. This process leads to the formation of two hydroxyl radicals (·OH).

(5) $$H_{2}O_{2}+h\nu \to 2(\cdot OH)$$

As detailed above, in photocatalytic systems based on ZnO nanorods, incident light promotes electrons from the VB to the CB, generating electron–hole pairs. The photogenerated holes in the VB possess strong oxidizing power. Hydroxyl radicals (·OH), which are highly reactive due to their unpaired electron, play a dual role in this process. One hydroxyl radical reacts with a photogenerated hole, effectively neutralizing it and thereby reducing electron–hole recombination. Concurrently, additional ·OH radicals react with pesticide molecules present on the sensor’s surface. For example, when a hydroxyl radical interacts with quinalphos, it initiates an oxidation reaction that yields degradation products, as shown in the reaction scheme below (Equation (6)) [26]. This dual functionality not only improves the charge separation efficiency of the ZnO nanorods but also facilitates the catalytic transformation of pesticide molecules, ultimately enhancing the sensor’s overall sensitivity and performance under illumination.

(6) $$\cdot OH+C_{12}H_{15}N_{2}O_{3}PS\to SO_{4}^{2-}+NO_{3}^{-}+PO_{4}^{3-}+H_{2}O+CO_{2}$$

When the hydroxyl radical reacts with thiamethoxam, the reaction gives the products mentioned below [27]:

(7) $$\cdot OH+C_{8}H_{10}ClN_{5}O_{3}S\to {\left(NH_{2}-NO_{2}\right)}^{-}+H^{2-}+{Cl}^{-}+NH_{3}^{-}+NO_{2}^{-}+S^{2-}$$

In Figure 6, it is evident that exposure to the quinalphos pesticide causes the formation of anionic species—specifically sulfate ($SO_{4}^{2-}$), nitrate ($NO_{3}^{-}$), and phosphate ($PO_{4}^{3-}$)—at the surface of the ZnO nanorods. These species act as electron donors by transferring electrons to the CB, thereby increasing the free carrier density. This additional electron injection enhances the conductivity of the ZnO nanorods, resulting in a reduction of the sensor’s resistance. Similarly, when the ZnO nanorods are exposed to thiamethoxam, there is an injection of several electron-donating species, including ${\left(NH_{2}-NO_{2}\right)}^{-}$, $H^{2-}$ (noting that the identification of these species may require further verification), chloride (${Cl}^{-}$), amide ($NH_{3}^{-}$), nitrite ($NO_{2}^{-}$), and sulfide ($S^{2-}$) ions. The donation of these ions further increases the electron density in the CB and results in a more pronounced decrease in resistance compared to that observed with quinalphos.

**Figure 7:**
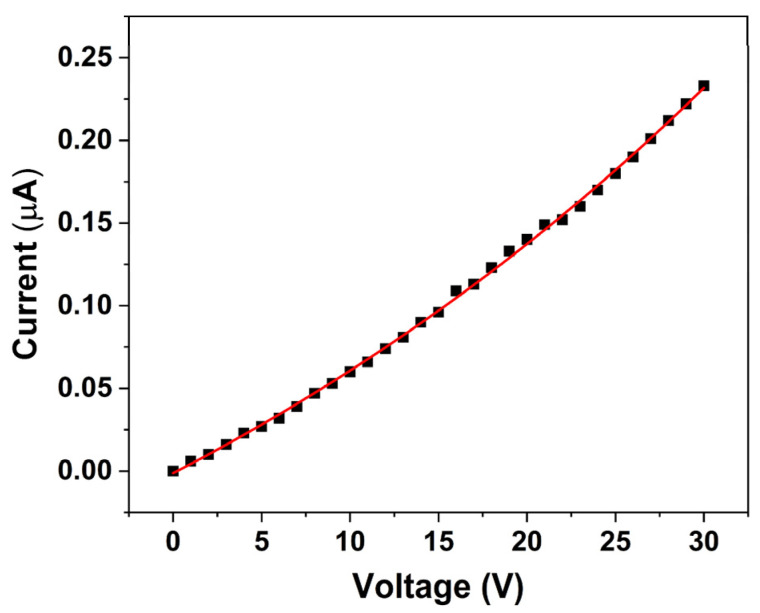
*I*–*V* characteristic of ZnO nanorod-based sensor acquired under visible light conditions. The square symbols represent experimental data, while the continuous line is the first-order exponential fitting curve.

The *I*–*V* characteristic of the ZnO nanorod-based sensor, shown in Figure 7, reveals that the sensor exhibits an initial resistance in the range of MΩs under room lighting conditions. Furthermore, the *I*–*V* relationship displayed in Figure 7 indicates that the sensor follows a first-order exponential behavior (Equation (8)):

(8) y=A1·exp(x/t1)+y0

Non-linear fitting of the experimental data yielded the following coefficients: y0 = −0.26744 µA, A1 = 0.2661 µA, and t1 = 0.02097 V, with an R^2^ value of 0.99922, indicating an excellent agreement between the experimental data and the fitted exponential equation. This non-linear response suggests that the conduction mechanism is dominated by processes such as charge carrier injection and transport, which are strongly modulated by the photoexcited electrons and surface defect states of ZnO. Overall, these observations underscore the critical role of surface charge transfer and photoexcitation in modulating the electrical properties of ZnO nanorods. The enhanced conductivity resulting from the donation of electrons by pesticide-induced species is fundamental to the sensor’s operation, ultimately enabling effective pesticide detection under ambient illumination conditions.

The sensor’s response was tested within the voltage range of 0 to 30 V, and a current range between 0.006 and 0.233 µA was determined.

### 3.4. Response of ZnO Nanorod-Based Sensor to Quinalphos

**Figure 8:**
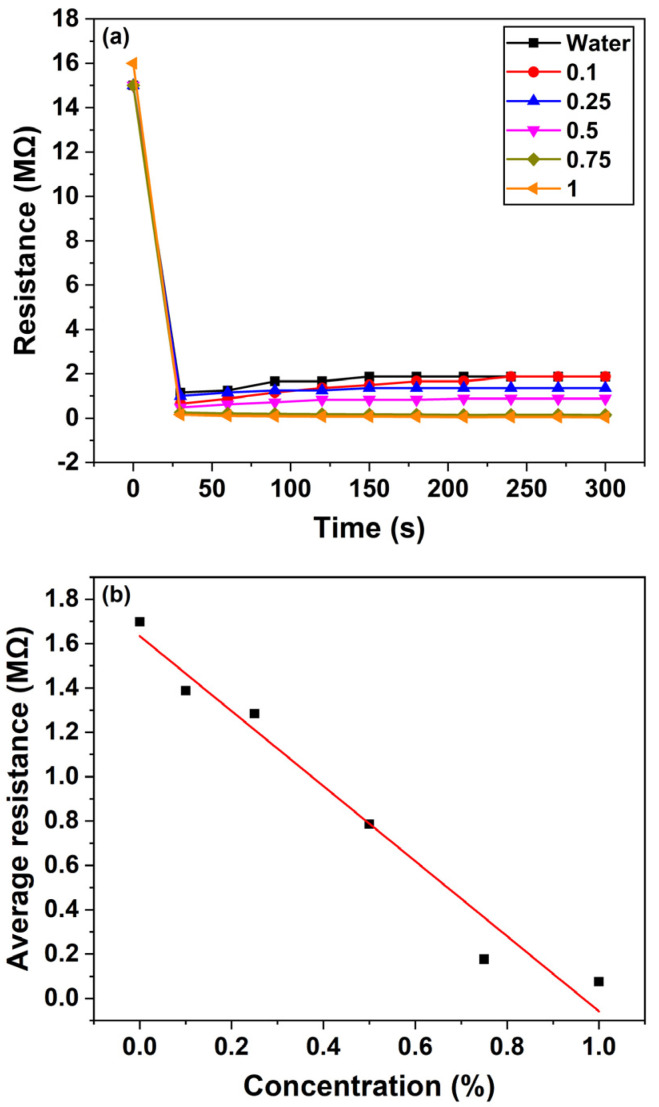
ZnO nanorod-based sensor’s response to quinalphos: (**a**) resistance change vs. time for a period of 5 min; (**b**) average resistance vs. concentration. The square symbols represent the experimental data, while the continuous line is the linear fitting curve.

Figure 8a depicts the temporal changes in the resistance of the ZnO nanosensor when exposed to various concentrations of the quinalphos pesticide. As the quinalphos concentration increases from 0.1 to 1% (*v*/*v*), the sensor’s resistance gradually decreases. This behavior suggests that electron-donating species released by quinalphos enhance the carrier density in the ZnO nanorods, thereby increasing the sensor’s conductivity. In experiments conducted over a duration of 5 min, the sensor’s resistance reached a steady state—or saturation—within approximately 30 s of exposure. This rapid stabilization indicates the sensor’s suitability for real-time monitoring applications.

Figure 8b illustrates the sensor’s average resistance change as a function of quinalphos concentration. In this graph, the *x*-axis represents the quinalphos concentration, expressed as a percentage (*v*/*v*), while the *y*-axis denotes the average resistance change in megohms (MΩ). The data reveal a linear decrease in the sensor’s resistance with increasing quinalphos concentration. The linear relationship is described by the calibration equation:

(9) y=1.63−1.69x

where y denotes the sensor’s resistance (in MΩ) and x represents the pesticide concentration (*v*/*v* percentage). The negative slope of −1.69 underscores the high sensitivity of the sensor, as a small increase in pesticide concentration results in a significant drop in resistance. Moreover, the R^2^ value of 0.96922 confirms an excellent linear correlation, demonstrating the consistency and reliability of the response for quantitative determination. More in detail, this calibration curve serves as a quantitative tool for determining the concentration of quinalphos in unknown samples, which is critical for quality assurance in agricultural and environmental applications. Overall, the rapid response and linear sensitivity of the ZnO nanosensor highlight its potential for efficient, real-time pesticide detection. The underlying mechanism—electron donation from pesticide-induced species leading to enhanced ZnO conductivity—forms the basis for its operational principle. These characteristics position the sensor as a promising candidate for integration into advanced monitoring systems that ensure environmental safety and food quality.

### 3.5. Response of ZnO Nanorod-Based Sensor to Thiamethoxam

**Figure 9:**
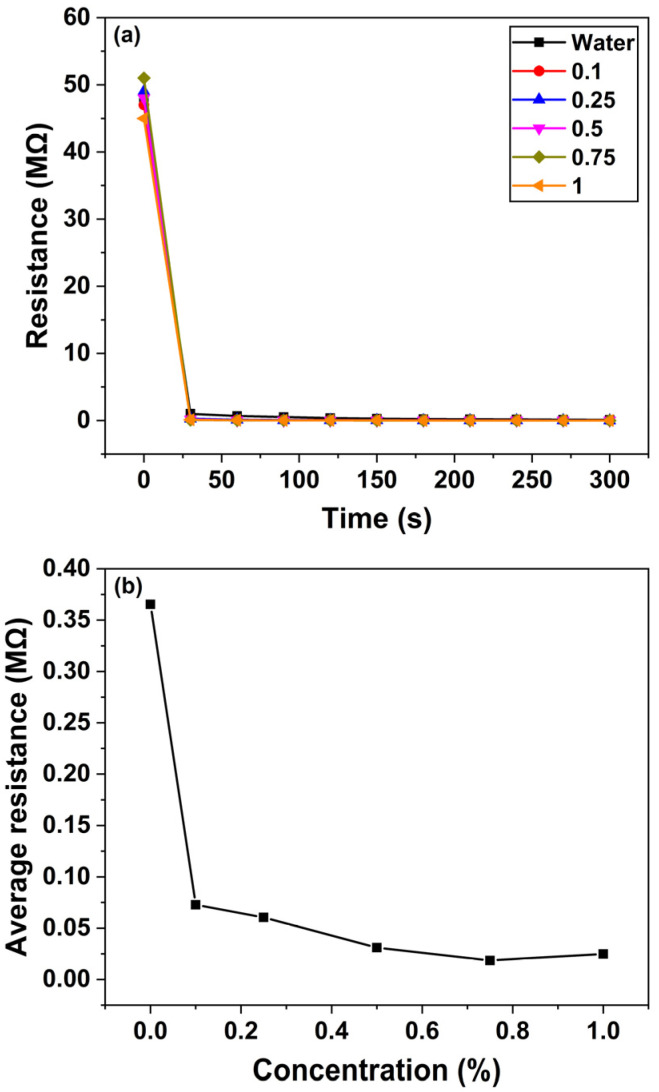
ZnO nanorod-based sensor’s response to thiamethoxam: (**a**) resistance change vs. time for a period of 5 min; (**b**) average resistance vs. concentration. The continuous line is just a guide to the eye.

Figure 9a illustrates the temporal response of the ZnO nanosensor’s resistance when exposed to various concentrations of the thiamethoxam pesticide. As the concentration of thiamethoxam increases from 0.1 to 1% (*v*/*v*), the sensor’s resistance gradually decreases. This decrease in resistance indicates that the conductivity of the ZnO nanorods increases due to the donation of electrons from thiamethoxam-related species to the CB. In experiments conducted over a 5 min interval, the sensor’s response was found to reach saturation within approximately 30 s, demonstrating a rapid and stable response suitable for real-time monitoring.

Figure 9b presents the average resistance change of the sensor as a function of thiamethoxam concentration. In this graph, the *x*-axis represents the pesticide concentration (expressed as a percentage, *v*/*v*), while the *y*-axis indicates the average resistance change in MΩ. The data reveal that as the thiamethoxam concentration increases, the sensor’s average resistance decreases; however, the relationship is non-linear. At higher concentrations, the resistance change begins to saturate, likely due to the limited number of active sites on the ZnO nanorods available for electron donation, or the onset of adsorption kinetics that deviate from ideal behavior.

This non-linear response suggests that while the sensor is highly sensitive to initial increases in thiamethoxam concentration, additional pesticide beyond a certain threshold does not produce a proportional decrease in resistance. The observed response is likely influenced by various factors such as the aggregation of adsorbed species, limited surface reaction sites, and competitive adsorption phenomena. Despite these complexities, the rapid saturation observed within 30 s underscores the potential of the ZnO nanosensor for efficient, real-time detection of thiamethoxam in practical applications. Overall, the distinct changes in sensor’s resistance with varying pesticide concentrations not only confirm the effective interaction between thiamethoxam and the ZnO nanorods but also provide a basis for further calibration and optimization of the sensor. This capability is essential for developing robust, portable devices for continuous environmental monitoring and for ensuring food safety.

The response of the sensor (SR%) to pesticide samples was determined based on the equation given below [25]:

(10) $$SR\%=\frac{\left(R_{no\;pesticide}-R_{pesticide}\right)}{R_{no\;pesticide}}\times 100$$

where $R_{no\;pesticide}$ and $R_{pesticide}$ are the resistances of the sensor in the absence and in the presence of pesticide, respectively.

Table 1 compares the response of the ZnO nanorod-based sensor to different concentrations of quinalphos and thiamethoxam pesticides in water. The results demonstrate that as the concentration of pesticide in water increases, the sensor’s response also increases for both quinalphos and thiamethoxam. In the case of quinalphos, the sensor exhibits a greater change in response with increasing pesticide concentration compared to thiamethoxam. More specifically, the sensitivity for quinalphos shifts from 90.32% at 0.1% (*v*/*v*) to 99.49% at 1% (*v*/*v*). For thiamethoxam, the sensitivity increases from 99.77% at 0.1% (*v*/*v*) to 99.97% at 1% (*v*/*v*). These distinct response profiles indicate that the sensor is capable of distinguishing between the two pesticides, thereby demonstrating its selectivity.

To evaluate the repeatability of the sensor’s response, two separate sensors were employed—one for each pesticide—with initial resistances of 4.3 and 14.1 MΩ for quinalphos and thiamethoxam, respectively. Prior to each subsequent measurement, the sensors were rinsed with water, and it was confirmed that the output resistance returned to its initial value. This step ensured that the sensors maintained a consistent baseline between tests.

**Figure 10:**
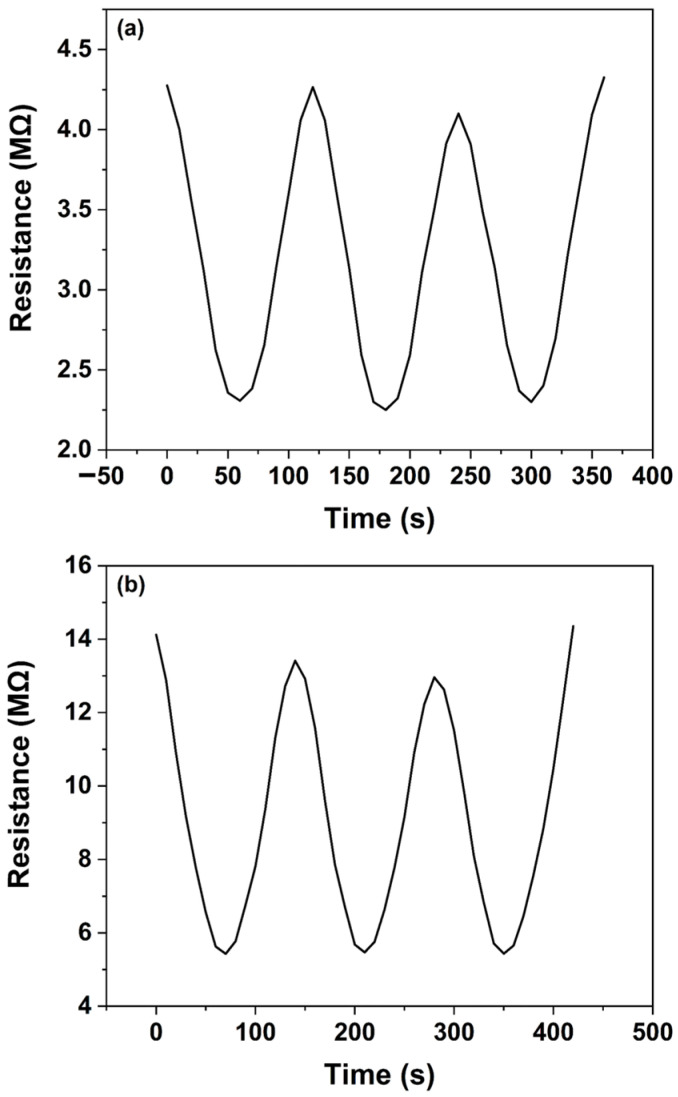
Repeatability test of the sensor for: (**a**) quinalphos and (**b**) thiamethoxam pesticides.

Figure 10a,b illustrate the repeatability and reproducibility of the sensor’s performance when the water sample contains 1% (*v*/*v*) of the quinalphos and thiamethoxam pesticides, respectively. The results confirm that the sensor not only exhibits a reliable response upon repeated exposures but also maintains its selectivity even at higher pesticide concentrations. Overall, the observed increase in the sensor’s response to higher pesticide concentrations, along with the distinct sensitivity profiles for quinalphos and thiamethoxam, underscores the potential of the ZnO nanosensor as a robust tool for environmental monitoring. The ability of the sensor to rapidly return to baseline and provide reproducible measurements is essential for practical applications in detecting and quantifying pesticide residues in water.

## 4. Discussion

### 4.1. Sensor’s Mechanism, Performance, and Selectivity

The present study investigated the development and performance of a ZnO nanorod-based sensor aimed at detecting pesticide residues, specifically quinalphos and thiamethoxam, in tea leaves. The results strongly indicate that the unique properties of ZnO nanorods—such as their high surface-to-volume ratio, efficient charge transport, and excellent chemical stability—are effectively harnessed to create a sensor exhibiting outstanding sensitivity, selectivity, and rapid response. This work elaborates on the underlying sensing mechanism, compares the sensor’s performance with traditional analytical techniques, and highlights both the potential applications and current limitations.

To highlight the performance advantages and novelty of the proposed ZnO nanorod-based sensor, Table 2 presents a comparative overview of recent ZnO-based sensors developed for pesticide detection, including their material composition, target analytes, key benefits, and reported innovations.

The intrinsic advantage of ZnO nanorods lies in their nanostructured morphology, which significantly increases the active surface area available for pesticide adsorption. According to the results, when pesticide molecules contact the ZnO nanorods, they are adsorbed onto the surface, resulting in variations in the local charge distribution. This alteration in surface charge leads to measurable changes in the sensor’s electrical conductivity. The enhanced sensitivity of the sensor, as demonstrated by a clear, reproducible correlation between pesticide concentration and the electrical response, is primarily due to the increased number of interaction sites available on the nanorods compared to bulk materials. Our experiments confirmed that even at low pesticide concentrations, the electrical signal from the sensor showed a detectable shift, thereby affirming the sensor’s capability for early warning applications in agricultural settings.

Further supporting the electrical measurements, UV–Vis spectroscopic analyses provided additional insights into the sensor’s functioning. The observed shift in the absorbance spectra, in accordance with the Beer–Lambert law, underscores the effectiveness of the ZnO nanorods in capturing pesticide molecules. By optimizing the sensor’s optical parameters, an optimal wavelength at which changes in absorbance directly correlated with pesticide concentration was determined. This dual modality—combining both electrical and optical detection—enhances the robustness of the sensor as it provides two independent avenues for quantification. The optical data not only validate pesticide adsorption but also offer a complementary technique to monitor real-time changes in the sensor’s environment during exposure.

A particularly noteworthy aspect of the sensor’s performance is its selectivity in complex matrices. The experimental results indicate that the ZnO nanorod sensor can discern quinalphos and thiamethoxam from other organic compounds commonly present in tea leaves. This selectivity was achieved by carefully controlling nanorod synthesis parameters such as precursor concentration and hydrothermal growth temperature, ensuring a uniform morphology and proper surface termination. The resulting sensor was not only able to distinguish between the targeted pesticides but was also largely unaffected by the presence of potential interferents. Such selectivity is critical in field applications where a myriad of organic substances may otherwise compromise detection accuracy.

Rapid sensor response represents another significant breakthrough as demonstrated by our kinetic studies. The sensor exhibited near-immediate changes in electrical conductivity upon exposure to the pesticides, indicating that adsorption dynamics and charge transfer occur on very short timescales. This rapid response is crucial for real-time monitoring applications, as it allows for prompt identification of potentially hazardous pesticide levels and facilitates timely intervention to mitigate contamination. In contrast, conventional analytical techniques such as GC and HPLC require lengthy sample preparation and processing times, underscoring the practical advantage of our sensor’s real-time capabilities.

The role of hydroxyl radicals in the sensor’s detection mechanism further enhances its functionality. Under light irradiation, ZnO nanorods generate electron–hole pairs that lead to the formation of reactive oxygen species, chiefly hydroxyl radicals. These radicals can actively oxidize pesticide molecules, thereby not only facilitating their detection by altering the surface properties of the sensor but also initiating their partial degradation. This unique dual functionality—simultaneous sensing and degradation—positions the ZnO nanorod sensor as a potential candidate for integrated environmental monitoring and remediation systems. Although the primary goal of this research was detection, the incidental observation of pesticide degradation opens new avenues for multifunctional sensor platforms capable of reducing environmental pollutant loads.

### 4.2. Practical Limitations and Future Directions

Despite these promising results, several limitations must be acknowledged. Our laboratory experiments were conducted under controlled conditions, and the sensor’s performance in variable field conditions—where factors such as humidity, temperature fluctuations, and complex chemical backgrounds are prevalent—remains to be thoroughly examined. Long-term stability is another key concern; although initial tests indicated good durability, extended field trials are necessary to ensure the sensor maintains its performance over time without significant degradation. Addressing these challenges will be critical for transitioning the sensor from a laboratory prototype to a widely implementable tool for routine environmental monitoring.

It is important to emphasize that the degradation pathway proposed in Figure 6 is theoretical, and derived from well-established mechanisms of ZnO-mediated photocatalysis. Although our electrical measurements, supported by the relevant literature, strongly suggest the generation of reactive oxygen species (ROS) and the subsequent degradation of pesticide molecules, direct experimental identification of the resulting degradation products was not conducted in this study. To address this, future work will focus on employing fluorescence-based probes and electron paramagnetic resonance (EPR) spectroscopy to detect and quantify ROS and intermediate species. These advanced characterization techniques will enable a more precise correlation between the observed resistance changes and the underlying charge carrier dynamics.

To summarize, the ZnO nanorod-based sensor developed in this study represents a considerable advancement in the detection of pesticide residues in tea leaves. Its high sensitivity, selectivity, and rapid response time, combined with its potential for dual sensing and remediation functions, make it a promising alternative to traditional pesticide detection methods. By providing real-time monitoring capabilities with lower operational costs and enhanced portability, this sensor is particularly suited for on-site applications in agricultural settings. Future research should focus on optimizing the sensor’s performance under variable environmental conditions and integrating it with wireless data transmission systems to facilitate a distributed monitoring network. Ultimately, the successful deployment of such advanced nanomaterial-based sensors could play a vital role in ensuring food safety and protecting public health.

### 4.3. Strengths, Weaknesses, Opportunities, and Threats (SWOT) Analysis

#### 4.3.1. Strengths

The proposed sensor exhibits high sensitivity and selectivity, demonstrating pronounced and distinguishable resistance changes in response to both quinalphos and thiamethoxam, even at low concentrations (as low as 0.1% *v*/*v*). Its rapid response time—stabilizing within approximately 30 s—makes it highly suitable for real-time monitoring applications. Furthermore, the sensor shows excellent repeatability and reproducibility, with minimal signal drift observed across multiple trials. Its portable and scalable design also enables seamless integration with field-deployable platforms, positioning it as a strong candidate for on-site pesticide detection in tea plantations.

#### 4.3.2. Weaknesses

A notable limitation is the non-linear response of the sensor to thiamethoxam, with saturation effects occurring at higher concentrations, which restricts its utility for precise linear calibration. Although the sensor displays selectivity toward the target pesticides, potential interference from chemically similar substances or complex organic matrices in real-world samples may affect detection accuracy under field conditions.

#### 4.3.3. Opportunities

This sensing platform holds significant potential for agricultural and environmental monitoring, particularly in tea plantations and other crop systems requiring in situ pesticide residue detection. Additionally, its compatibility with Internet of Things (IoT) architectures offers a promising avenue for remote, wireless data transmission and large-scale deployment in distributed sensing networks.

#### 4.3.4. Threats

Despite its advantages, the sensor competes with established and highly validated analytical techniques such as GC–MS and HPLC, which remain the gold standard in regulatory pesticide testing. These methods, while more resource-intensive, may still be preferred in official monitoring programs due to their broader acceptance and validation.

## 5. Conclusions

This study presented the development and comprehensive evaluation of a ZnO nanorod-based sensor designed for the detection of the quinalphos and thiamethoxam pesticides, which are frequently employed in tea gardens. The sensor functions through a change in resistance upon exposure to the pesticides. For quinalphos, an increase in concentration resulted in a gradual decrease in the sensor’s resistance, which can be quantitatively described by the linear equation y=1.63−1.69x, where y represents the sensor’s resistance and x denotes the pesticide concentration. In the case of thiamethoxam, the resistance also decreased with increasing concentration, although the response presented a saturation behavior at higher concentrations. Maximum sensor responses of 99.49% for quinalphos and 99.97% for thiamethoxam were achieved at room temperature with a minimum detection limit of 0.1% (*v*/*v*) in water. Furthermore, repeatability tests confirmed that the sensor returns reliably to its baseline resistance after each measurement, demonstrating excellent reproducibility and selectivity for both pesticides. Overall, the ZnO nanorod-based sensor exhibited rapid response, high sensitivity, and robust performance. These characteristics affirm its potential for real-time monitoring of pesticide residues in agricultural environments, thereby supporting quality control and environmental safety in the tea industry. Future work will focus on further optimizing the sensor’s performance by testing under light of different wavelengths and intensities and exploring its integration into portable systems for on-site analyses.

## Figures and Tables

**Table 1:** Response of the ZnO sensor to different concentrations of quinalphos and thiamethoxam pesticides in water.

Concentration of Pesticide in Water (%)	SR% to Quinalphos	SR% to Thiamethoxam
0.1	90.32	99.77
0.25	91.44	99.95
0.5	94.76	99.97
0.75	98.82	99.97
1	99.49	99.97

**Table 2:** Comparison of recent ZnO-based sensors for pesticide detection in terms of materials used, target analytes, reported advantages and claimed novelty.

No.	ZnO Material/System	Target Pesticides	Advantages	Novelty	Ref.
1	ZnO nanostructures directly grown on flexible substrates (carbon paper and carbon cloth)	Organophosphates such as paraoxon	- High sensitivity - Low limit of detection (LOD): 0.5 nM–5 µM - Good operational stability	- Biosensors were developed by immobilizing the acetylcholinesterase (AChE) enzyme onto ZnO nanostructures - This configuration enhances enzyme activity and electron transfer, contributing to the sensor’s high performance	[28]
2	3-amminopropyltrimethoxysilane (APTES)-capped ZnO quantum dots (QDs)	Aldrin, Tetradifon, Glyphosate, Atrazine	- Dual-mode sensing: optical (fluorescence quenching) and electrochemical (resistance change) - High binding affinities (*K*_b_ ≈ 10^7^–10^13^ M^−1^) - Distinct sensing signatures enable effective pesticide differentiation - QDs also act as photocatalysts	- Detailed analysis of fluorescence behavior, lifetime, and binding interactions between ZnO QDs and structurally diverse pesticides - First study to utilize electrochemical resistance changes of ZnO QDs for selective detection of these pesticides - Combined optical-electrochemical sensing strategy for improved pattern recognition and analyte discrimination	[29]
3	Semiconductor-based nanomaterials, such as ZnO nanowires	Common grape pesticides	High sensitivity, low detection limits, rapid and real-time response, label-free detection, potential for multiplexing, and suitability for miniaturization and portable applications	- Utilization of nanostructured ZnO and other advanced nanomaterials for direct, on-site, and real-time detection of multiple pesticide residues in grapes - Seamless integration into portable and IoT-enabled sensing platforms	[30]
4	ZnO-based nanomaterials	Organochlorine pesticides (OCPs)	- Development of low-cost, user-friendly, and portable sensing devices - Capable of detecting pesticides at trace levels with high sensitivity - Rapid alternative to conventional, complex, and time-intensive analytical methods - ZnO nanomaterials also investigated for their potential in pesticide degradation	Comprehensive review highlighting recent advancements in ZnO-based electrochemical sensors for OCP detection, with emphasis on performance metrics (LOD, linear range), fabrication strategies, and real-world applicability	[31]
5	ZnO nanoparticles (sonochemically synthesized) + mixed cellulose acetate (MCA) polymeric membrane filtration	Organophosphates such as chlorpyrifos	- Enhanced degradation efficiency compared to ZnO photocatalyst alone - Efficient degradation (max at 60 min for 5 ppm) - Recovery and potential reuse of ZnO photocatalyst - Time-efficient approach for pesticide degradation	Integration of photocatalysis using synthesized ZnO nanoparticles with membrane filtration in a photocatalytic membrane filtration reactor (PMFR) enables efficient pesticide degradation alongside simultaneous recovery of the catalyst	[32]
6	ZnO nanoparticles drop-cast on the surface of a disposable screen-printed carbon electrode (SPCE)	Glyphosate	- High sensitivity (LOD: 0.648 µM in buffer, 0.96 µM in river water) - Wide linear range (0.5 µM–7.5 mM) - Excellent selectivity against common interferents - Rapid detection (30 min incubation) - Cost-effective, disposable format - Eco-friendly synthesis	- Solid-state electrochemical sensor using ultra-small (~7 nm) ZnO nanoparticles produced via green synthesis (lemon extract) for sensitive and selective on-site glyphosate detection - Strong Zn-glyphosate interaction mechanism confirmed by DFT calculations	[33]
7	ZnO nanoparticles and nanorods	Quinalphos and thiamethoxam (in tea leaves)	- High sensitivity and selectivity - Rapid response (~30 s) - Excellent repeatability and reproducibility - Portable and low-cost fabrication - Real-time on-site capability	First report of ZnO nanorod-based chemiresistive sensor for simultaneous detection and discrimination of quinalphos and thiamethoxam in tea leaves - Distinct linear (quinalphos) and non-linear (thiamethoxam) response mechanisms - Dual function of sensing and partial degradation under visible light	This work

## Data Availability

Data are available on request due to restrictions of privacy.

## Abbreviations

The following abbreviations are used in this manuscript:

AChE	Acetylcholinesterase
APTES	3-amminopropyltrimethoxysilane
CB	Conduction band
DC	Direct current
DFT	Discrete Fourier transform
DI	Deionized
EPR	Electron paramagnetic resonance
GC	Gas chromatography
GC–MS	Gas chromatography–mass spectrometry
HPLC	High-performance liquid chromatography
IoT	Internet of things
*I*–*V*	Current–voltage
JCPDS	Joint Committee on Powder Diffraction Standards
LC–MS	Liquid chromatography–mass spectrometry
LED	Light-emitting diode
LOD	Limit of detection
MCA	Mixed cellulose acetate
OCP	Organochlorine pesticide
PCB	Printed circuit board
PMFR	Photocatalytic membrane filtration reactor
QD	Quantum dot
ROS	Reactive oxygen species
SEM	Scanning electron microscopy
SPCE	Screen-printed carbon electrode
SWOT	Strengths, Weaknesses, Opportunities, and Threats
TEM	Transmission electron microscopy
UV–Vis	Ultraviolet–visible
VB	Valence band
XRD	X-ray diffraction
